# Preparation of a low-cost and eco-friendly superabsorbent composite based on wheat bran and laterite for potential application in Chinese herbal medicine growth

**DOI:** 10.1098/rsos.180007

**Published:** 2018-05-23

**Authors:** Jiande Gao, Jin Liu, Hui Peng, Yaya Wang, Sha Cheng, Ziqiang Lei

**Affiliations:** 1 Key Laboratory of Eco-Environment-Related Polymer Materials of Ministry of Education, Key Laboratory of Polymer Materials of Gansu Province, College of Chemistry and Chemical Engineering, Northwest Normal University, Lanzhou 730070, People's Republic of China; 2 College of Pharmacy, Gansu University of Traditional Chinese Medicine, Lanzhou 730000, People's Republic of China

**Keywords:** laterite, superabsorbent composite, urea fertilizer carrier, Chinese herbal medicine growth

## Abstract

A low-cost and eco-friendly superabsorbent composite is prepared through the free-radical graft co-polymerization of wheat bran (WB), acrylic acid (AA) and laterite (LA) in an aqueous solution. Elemental map, scanning electron microscopy and Fourier transform infrared spectra revealed that the LA evenly distributed in the superabsorbent composite and wheat bran-g-poly(acrylic acid)/laterite (WB-g-PAA/LA) formed successfully. Thermogravimetric analysis confirmed that the WB-g-PAA/LA had high thermal stability. Furthermore, the properties of the WB-g-PAA/LA, such as swelling in saline solutions and degradation, are also assessed. The final WB-g-PAA/LA (5 wt%) superabsorbent composite attained an optimum water absorbency of 1425 g g^−1^ in distilled water and 72 g g^−1^ in 0.9 wt% NaCl solution. The water absorbency of WB-g-PAA/LA (10 wt%) is even greater than that of the WB-g-PAA. Moreover, the water-retention capacity of WB-g-PAA/LA (5 wt%) is high, and the water-retention process followed a zero-order reaction. The reaction rate constant is 8.2428 × 10^5^ exp(−*E*_a_*/RT*) and the apparent activation energy (*E*_a_) is 35.11 kJ mol^−1^. Furthermore, WB-g-PAA/LA (5 wt%) may regulate the release of urea, indicating that the superabsorbent composite could provide a promising application as a urea fertilizer carrier. Additionally, it increased the germination and growth rates of *Glycyrrhiza uralensis* Fisch, suggesting it could influence the growth of Chinese herbal medicine.

## 1. Introduction

Fertilizer and water play critical roles in increasing crop yields [1]. However, application of large amounts of fertilizer has already caused serious environmental contamination and economic loss [2]. Water is another significant factor limiting agricultural development [3]. Crop growth and sustainable ecosystems are restricted owing to water shortages in arid and semi-arid regions, while most rainwater is lost and not used efficiently during rainfall in these regions. Under such circumstances, a new strategy for saving fertilizer and water involves the utilization of superabsorbent composites. Superabsorbent composites represent a class of hydrophilic polymer materials with lightly cross-linked network structures, which can absorb and hold a great deal of water or other aqueous solutions. Moreover, superabsorbent composites can relieve water evaporation from the soil in dry and hot climates and increase soil fertility [4]. Because of these advantages, superabsorbent composites have evoked a strong research desire in agriculture and horticulture [5]. Currently, the main raw materials used in preparing commercial superabsorbents are obtained from petrochemical products, including acrylamide, acrylic acid (AA) and acrylonitrile. As the petrochemical products are costly, poorly degradable, non-renewable and environmentally unfriendly, the practical application of the petroleum-based superabsorbents has been severely restricted [6]. To overcome these drawbacks, renewable natural resources, especially agricultural wastes, such as pineapple peel [7], peanut hull [8], wheat straw [9] and corn straw, [10] have attracted considerable research attention owing to their economic efficiency and environmental friendliness. Simultaneously, the incorporation of low-cost clays, such as kaolin, [11] attapulgite, [12] illite and smectite, [13] into polymer networks, to reduce final production costs, enhance the swelling properties of superabsorbent composites, and supply a slower and more continuous release mode, has become a novel strategy in the development of superabsorbent composites [14–16]. While many reports regarding agricultural waste-based superabsorbent composites have mainly focused on their properties, including swelling and de-swelling behaviours, a few reports provided systematical research on their application as fertilizer carriers or their effects on the growth of Chinese herbal medicine [17,18]. Actually, development of a novel environmentally friendly and low-cost superabsorbent composite is expected, and its application in new fields is also much desired.

Wheat bran (WB) is an agricultural waste from wheat flour processing, which contains high contents of cellulose, starch and protein. More than 10 million tons of WB could be produced per year in China [19]. Only minor amounts are used for food purposes and mostly for livestock feed. Although there were reports on WB being used as a raw material in various industries, its utilization efficiency was still not able to meet expectations [20]. Thus, it is necessary for WB to be developed and applied in a simple and efficient way. Laterite (LA), which may contain a lot of quartz and kaolinite, [21] is a porous and coarse clay with many reactive hydroxyl functional groups (–OH) on its surface, [22] and is found abundantly in tropical regions. The reports on LA applications are mainly devoted to its use in adsorbents; little information could be found regarding LA as a filler material in polymer networks [23,24]. In our previous study, the superabsorbent composites based on raw WB and clays were developed, which mainly concentrated on the effects of clay types on the properties of the wheat bran-g-poly(acrylic acid)/clays (WB-g-PAA/clays) using the same clay content and reaction conditions [25]. As a result, LA played a striking role in some properties for superabsorbent composites. The properties and costs of superabsorbent composites might not be encouraging due to the contents of WB and LA not being adequately researched. This work is to focus on turning WB and LA into low-cost, high-performance and eco-friendly superabsorbent composites, and investigating the application of superabsorbents in urea fertilizer carriers and in the growth of Chinese herbal medicine. Specific objectives included: (a) optimization of conditions to prepare the WB-g-PAA/LA superabsorbent composite; (b) studying the properties of the WB-g-PAA/LA and (c) investigating its applications as a urea fertilizer carrier and in the growth of a Chinese herb.

## 2. Experimental

### 2.1. Materials

Sodium alginate (SA) was purchased from Shanghai Chemical Co. Ltd, China. Acrylic acid (AA, analytical grade) was purchased from Tianjin Kaixin Chemical Industrial Co., China. *N*,*N*'-methylenebisacrylamide (MBA, analytical grade) was purchased from Sinopharm Chemical Reagent Co., Ltd, China. Potassium persulfate (KPS, analytical grade) was purchased from Tianjin Kaitong Chemical Industrial Co., China. WB was obtained from a flour mill (Jingyuan, Gansu Province, China) and dried in an oven at 80°C to a constant weight and further passed through a 160-mesh sieve before use. LA was collected from WuQuan Mountain (Lanzhou, Gansu Province, China), dried in an oven at 105°C for 24 h and sieved through a 200-mesh screen before use. All other reagents used were of analytical grade, and all solutions were prepared with distilled water.

### 2.2. Preparation of superabsorbent composites

A series of the WB-g-PAA/LA superabsorbent composites were prepared according to the following procedure: distilled water (25 ml) and WB (2.0 g) were added to a 250 ml three-necked flask equipped with a mechanical stirrer. The three-necked flask was placed in a thermostat water bath preset at 70°C, with stirring for 30 min to form a homogenized mixture. Then, the mixture was cooled to 50°C, and the weighed initiator KPS was added and stirred for an additional 15 min. After that, weighed quantities of the cross-linker MBA, LA powder and AA monomer, with a 70% neutralization degree, were incorporated into the mixture. The reaction temperature was allowed to increase to 70°C and was maintained for 1.5 h to complete polymerization. Nitrogen atmosphere protection was maintained throughout the reaction period. Finally, the resulting samples were dried in an oven at 60°C to constant weights and milled using a pulverizer prior to use. The preparation procedure of WB-g-PAA was similar to that of the WB-g-PAA/LA, except without LA being added.

### 2.3. Preparation of urea bead-coated superabsorbent composites

The urea bead-coated superabsorbent composites (UBCSCs) were prepared according to the following procedure: firstly, urea (60 g), LA (5 g) and distilled water (100 ml) were added to a 250 ml flask under mechanical stirring (200 rpm) for 30 min. Subsequently, 3 g of SA was slowly added with further stirring for 2 h to form a homogeneous mixture. Next, the mixture solution was dropped into a 5 wt% CaCl_2_ solution using a 1 ml syringe. Urea beads (UBs) were formed immediately because SA was cross-linked by the Ca^2+^ in the 5 wt% CaCl_2_ solution, which exhibits high mechanical strength through SA gel beads and a viscous lateritic clay after drying. The ratio of the mixed solution and 5 wt% CaCl_2_ solution was 1 : 4 (*V*/*V*). The obtained UBs (approx. 5 mm in diameter in their swollen state) were filtered and placed into a rotating pan to coat with WB-g-PAA/LA superabsorbent composite (below 110 mesh). In this way, the UBCSC was obtained with WB-g-PAA/LA (5 wt%) adhered to the outer surface of the UBs. Finally, the UBCSC was dried at room temperature to obtain the final product. The content of nitrogen in the UBCSC was determined using an elemental analysis instrument (Germany Elemental Vario EL Corp., model 1106), and the content was 26.15 wt%.

### 2.4. Evaluation of properties

#### 2.4.1. Degradation of wheat bran-g-poly(acrylic acid)/laterite in soil supernatant

To investigate the degradation of WB-g-PAA/LA (5 wt%), a soil supernatant was prepared by the centrifugation separation method in a manner similar to that reported by Davies & Davies [26]. First, 200 g of soil was immersed in 1000 ml of distilled water for 24 h. Then, the sample was centrifuged to obtain the soil supernatant. The pre-weighed WB-g-PAA/LA (5 wt%) slice (approx. 8 mm in diameter and 2 mm in thickness) was immersed in 50 ml of the soil supernatant at the ambient temperature. Nine replicates were performed. At the predetermined time, the dried slice was removed from the soil supernatant through a silk sieve (300 mesh), washed repeatedly with distilled water and dried at 60°C to a constant weight. The percentage of the degradation was calculated by measuring the weight loss using the following equation:

2.1 $$\text{degradation }(\%)=\frac{(W_{0}-W_{t})\times 100}{W_{0}},$$

where *W*_0_ and *W_t_* indicate the weights of superabsorbent discs before and after degradation, respectively.

To study the effect of incorporation of 5 wt% LA on the biodegradability of the superabsorbent composite, the degradation of WB-g-PAA was carried out under the same condition.

#### 2.4.2. Effects on urea release in soil

Release rates of urea nitrogen from UBCSCs were determined by burying 2.4 g of UBCSC granules, which were divided into eight sealed plastic mesh bags. The bags were buried approximately 5 cm below the surface in pots containing 200 g of dry soil (below 20 mesh, the same as described in the electronic supplementary material). Throughout the experiment, the soil moisture was maintained at 30% by weighing and adding tap water. The bags with UBCSCs granules were removed after each incubation period (2–4, 10, 15, 20, 25 and 35 days), and then dried at room temperature to a constant weight to determine the nitrogen contents.

For comparison purposes, the UB granules were investigated under the same conditions and used as controls.

## 3. Results and discussion

### 3.1. Fourier transform infrared spectral analysis

**Figure 1.**
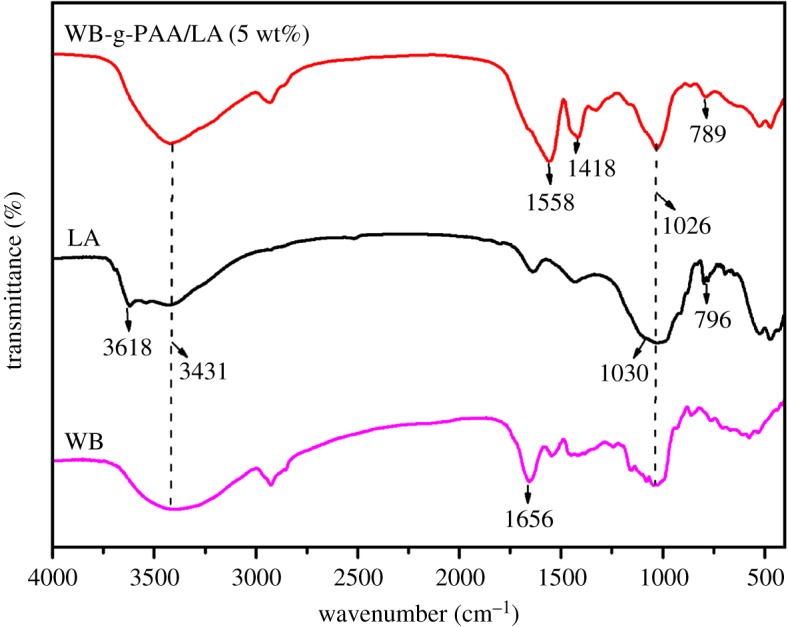
FTIR spectra of WB, LA and WB-g-PAA/LA (5 wt%).

The FTIR spectra for WB, LA and WB-g-PAA/LA (5 wt%) are shown in figure 1 (see detail parameters, electronic supplementary material, S2). The absorption bands between 3400 and 3620 cm^−1^ are attributed to the stretching vibration of −OH [27]. Compared with the FTIR spectrum of WB, the absorption band at 1656 cm^−1^ can be assigned to the stretching vibration of the −C=O in −COOH groups of the polysaccharide structure in the WB [28], but it almost disappears in the FTIR of WB-g-PAA/LA (5 wt%). However, two new absorption bands at 1558 and 1418 cm^−1^ appear in the FTIR of WB-g-PAA/LA (5 wt%), [29] which are ascribed to the asymmetric and symmetric stretching vibrations of –COO^−^ groups, respectively. The absorption band at 1026 cm^−1^ is attributed to the stretching vibration of C–O–C in the FTIRs of WB-g-PAA/LA (5 wt%) and WB. In the FTIR spectrum of LA, the absorption band at 3618 cm^−1^ corresponds to –OH stretching vibration, but it disappears in the FTIR of WB-g-PAA/LA (5 wt%). The absorption band at 1030 cm^−1^ is ascribed to Si–O–Si stretching vibration, which is obviously weakened in the FTIR of WB-g-PAA/LA (5 wt%). Additionally, the absorption band at 796 cm^−1^ in the FTIR of the LA is ascribed to the Si–O–Al stretching mode [30], with a slight shift to 789 cm^−1^ after reaction. All these variations of absorption bands demonstrate that the WB and LA participate in the graft co-polymerization reaction of WB-g-PAA/LA (5 wt%).

### 3.2. Morphological analysis

**Figure 2.**
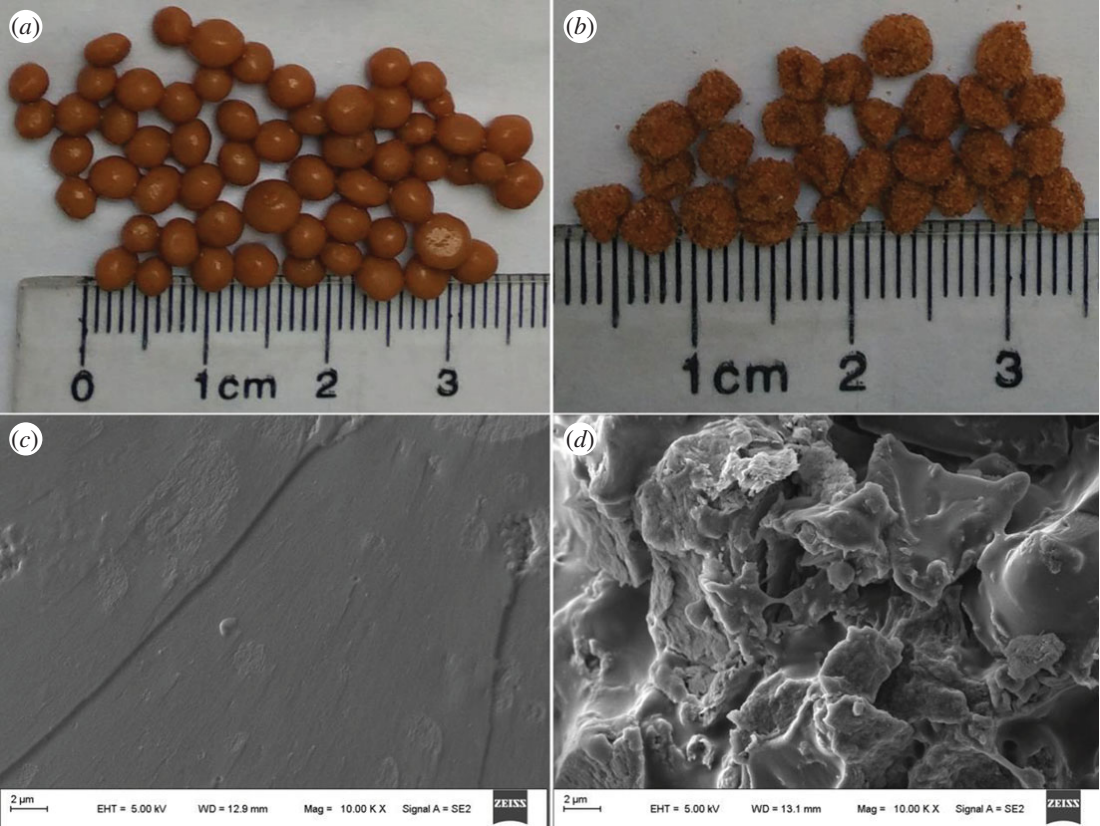
Digital photo and SEM image: (*a*) digital photo of UB granules in a swollen state, (*b*) digital photo of UBCSC granules before being dried, (*c*) SEM image of WB-g-PAA and (*d*) SEM image of WB-g-PAA/LA (5 wt%).

Figure 2 shows the digital photos of the UB granules and UBCSC granules, as well as scanning electron microscopic (SEM) images of WB-g-PAA and WB-g-PAA/LA (5 wt%) (see detail parameters, electronic supplementary material, S2). In figure 2*a*, swollen UB granules appear as brown spheres with smooth surfaces. However, the UBCSC granules have a rough surface with a diameter approximately of 5 mm (figure 2*b*). Additionally, it could be seen from the SEM images that WB-g-PAA shows a very tight, flat and smooth surface (figure 2*c*), while WB-g-PAA/LA (5 wt%) exhibits a loose and ruggedly fractured surface (figure 2*d*). These results indicated that the incorporation of 5 wt% LA can increase the porosity of the superabsorbent composite, which is beneficial for increasing the water absorbency of superabsorbent composites [31]. WB-g-PAA/LA (5 wt%) may also have better degradability than WB-g-PAA owing to the loose and ruggedly fractured surface in agricultural and horticultural application.

**Figure 3.**
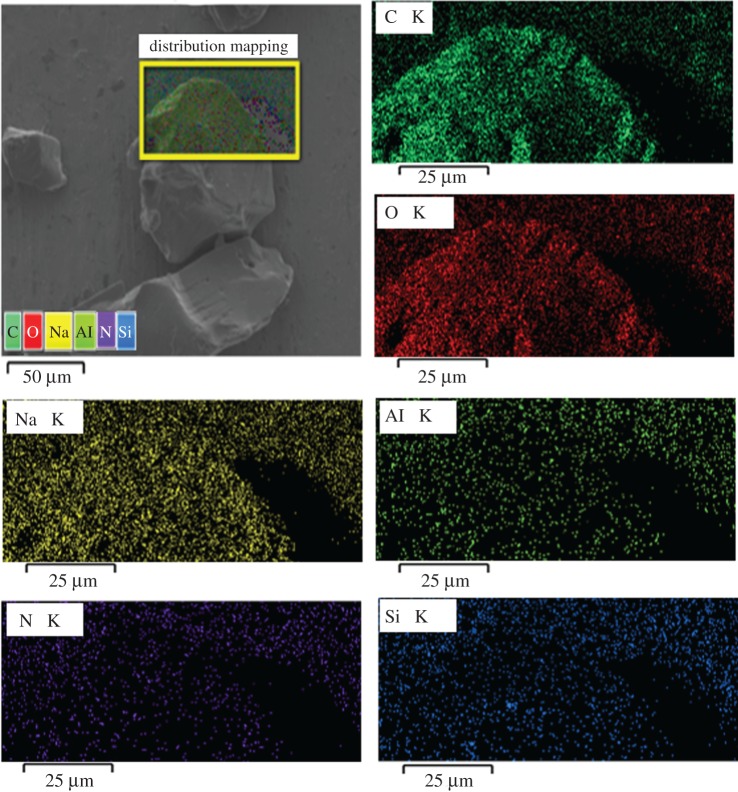
Elemental map of WB-g-PAA/LA (5 wt%).

To prove that the incorporation of 5 wt% LA is dispersed into the network of WB-g-PAA/LA, the elemental map (EM) for the WB-g-PAA/LA (5 wt%) sample is examined. It can be seen from figure 3 that the characteristic Na, Al, Si, N, C and O elements of LA clay exist in the EM of WB-g-PAA/LA (5 wt%), which indicate that 5 wt% LA is dispersed in the WB-g-PAA/LA (5 wt%) network evenly.

### 3.3. X-ray diffraction analysis

**Figure 4.**
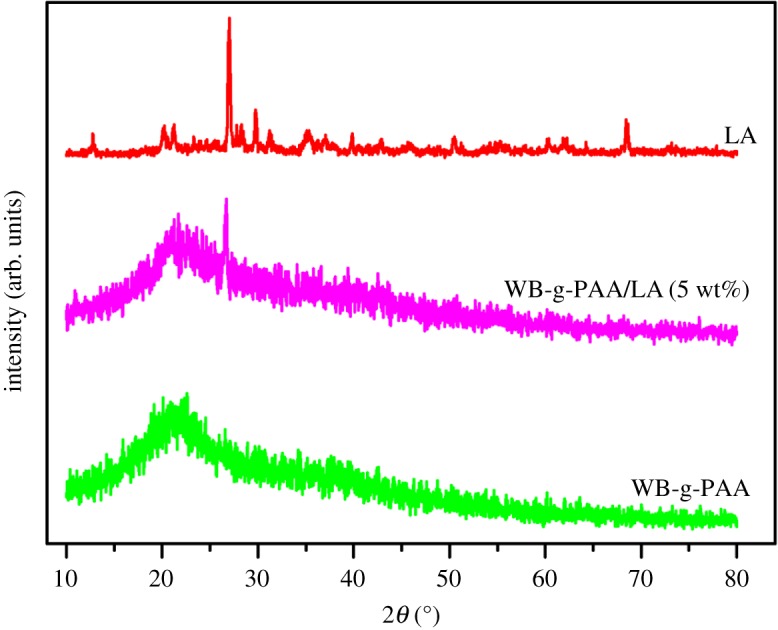
XRD patterns of LA, WB-g-PAA/LA (5 wt%) and WB-g-PAA.

The XRD patterns of LA clay, WB-g-PAA/LA (5 wt%) and WB-g-PAA are shown in figure 4 (see detail parameters, electronic supplementary material, S2). It can be observed that LA exhibits strong diffraction peaks at 2*θ* = 12.76°, 2*θ* = 21.24 and 2*θ* = 27.7°, which are ascribed to the characteristic diffraction of muscovite and SiO_2_. For WB-g-PAA/LA (5 wt%), the presence of the characteristic peak at 2*θ* = 27.04° confirmed the incorporation of LA, but the characteristic peak shifted and weakened in comparison to that of LA, implying that the LA is exfoliated during the reaction and uniformly dispersed in the superabsorbent composite. The XRD patterns of WB-g-PAA present typical amorphous structure with low crystallinity at 2*θ* = 22°. All the XRD results are consistent with the FTIR results.

### 3.4. Thermal stability analysis of wheat bran-g-poly(acrylic acid)/laterite (5 wt%)

**Figure 5.**
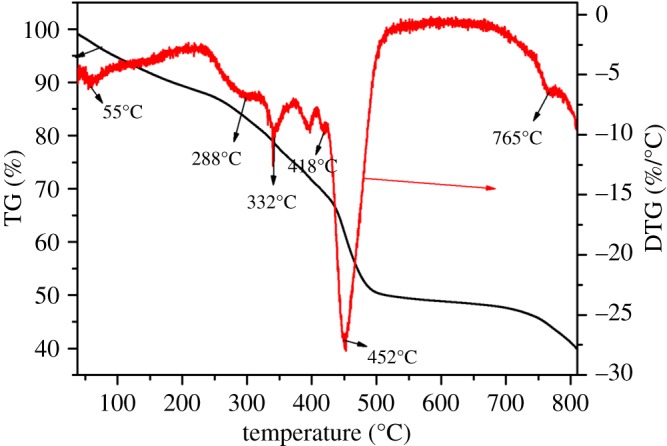
Thermal stability analysis of WB-g-PAA/LA (5 wt%).

The thermal stability curve of WB-g-PAA/LA (5 wt%) is depicted in figure 5 (see detail parameters, electronic supplementary material, S2). The thermal decomposition process of WB-g-PAA/LA (5 wt%) exhibited mainly three stages. At the initial stage, the weight loss of about 16.7% from room temperature to 288°C for WB-g-PAA/LA (5 wt%) is ascribed to the loss of volatile compounds or bound water. In the second stage, the weight loss of about 30.7% between 288 and 418°C for WB-g-PAA/LA (5 wt%) is associated with the dehydration of saccharide rings in WB and the elimination of H_2_O from the two neighbouring –COOH groups of the polymer chains [32]. In the last stage, WB-g-PAA/LA (5 wt%) was disintegrated between 418 and 800°C, which can be attributed to the destruction of the main chain and the cross-linked network structure [33].

### 3.5. Studies of reaction conditions

#### 3.5.1. Effect of the weight ratio of acrylic acid to wheat bran on water absorbency

**Figure 6.**
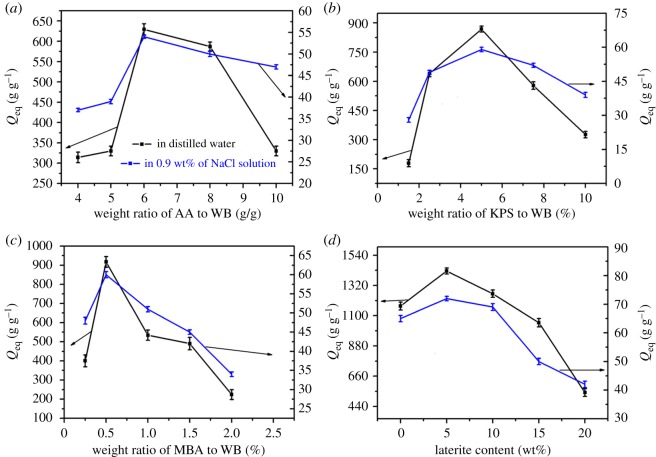
Effects of reaction conditions on water absorbency: (*a*) weight ratio of AA to WB, (*b*) weight ratio of KPS to WB, (*c*) weight ratio of MBA to WB and (*d*) LA content.

As shown in figure 6*a*, the weight ratio of AA to WB had a great influence on the water absorbency of WB-g-PAA/LA (reaction conditions: weight ratio of KPS to WB, 7.5%; weight ratio of MBA to WB, 1.5%; neutralization degree of AA, 70%; LA content, 2 wt%; reaction time 1.5 h; reaction temperature 70°C). The water absorbency initially increased and then decreased as the weight ratio of AA to WB increased. When the weight ratio of AA to WB was 6 g g^−1^, the maximum water absorbency of WB-g-PAA/LA that can be reached is 629 g g^−1^ in distilled water and 54 g g^−1^ in 0.9 wt% of NaCl solution. The water absorbency was decreased when the weight ratio of AA to WB was greater than 6 g g^−1^ because the superfluous AA monomer could form a PAA homopolymer, which increased the viscosity of reaction system and eventually inhibited the reaction, leading to the decreased water absorbency level [34]. When the weight ratio was lower than 6 g g^−1^, there were fewer hydrophilic functional groups, including −COO^−^ and −COOH in the polymer network, causing a lower water absorbency level [35].

#### 3.5.2. Effect of the potassium persulfate to wheat bran weight ratio on water absorbency

The effect of the KPS to WB weight ratio on water absorbency was investigated in detail (reaction conditions: weight ratio of AA to WB, 6 g g^−1^; weight ratio of MBA to WB, 1.5%; neutralization degree of AA, 70%; LA content, 2 wt%; reaction time 1.5 h; reaction temperature 70°C); the results are shown in figure 6*b*. With the increase in the weight ratio of KPS to WB from 1.5 to 5%, the maximum water absorbency levels of 869 g g^−1^ and 58 g g^−1^ were obtained in distilled water and 0.9 wt% NaCl solution, respectively, but decreased with further increases in the KPS to WB weight ratio. With a smaller amount of KPS, the polymerization reaction may have been sluggish and incomplete, and the network structure in the polymer skeleton does not form efficiently, causing the low water absorbency. Higher weight ratios of KPS to WB resulted in a rate of polymerization that was too rapid to be controlled, forming a resin with lots of branched chains, which has a negative effect on water absorbency [36].

#### 3.5.3. Effect of the *N*,*N*'-methylenebisacrylamide to WB weight ratio on water absorbency

The effect of the MBA to WB weight ratio on water absorbency is shown in figure 6*c* (reaction conditions: weight ratio of AA to WB, 6 g g^−1^; weight ratio of KPS to WB, 5%; neutralization degree of AA, 70%; LA content, 2 wt%; reaction time 1.5 h; reaction temperature 70°C). Water absorbency sharply decreased as the weight ratio of MBA to WB increased from 0.5 to 2.0%, as in figure 6*c*. When the MBA to WB weight ratio was 0.5 wt%, the maximum water absorbency levels of 918 g g^−1^ and 60 g g^−1^ were achieved in distilled water and 0.9 wt% NaCl solution, respectively. With the increase in the MBA cross-linker, more cross-linking points were generated during the polymerization process, which caused a high cross-linking density. This decreased the amount of network space for holding water and thus caused the low water absorbency. However, a lower MBA to WB weight ratio caused an increase in the soluble material owing to the lower cross-linking density. As a result, the water absorbency decreased. This result was similar to that of a previous report [37].

#### 3.5.4. Effect of the laterite content on water absorbency

As shown in figure 6*d* (reaction conditions: weight ratio of AA to WB, 6 g g^−1^; weight ratio of KPS to WB, 5%; weight ratio of MBA to WB, 0.5%; neutralization degree of AA, 70%; reaction time 1.5 h; reaction temperature 70°C), the water absorbency of WB-g-PAA/LA increased with incorporation of LA from 0 to 5 wt% in WB-g-PAA and then gradually decreased with further increases from 5 to 20 wt% both in distilled water and in 0.9 wt% NaCl solution. A small amount of LA in the WB-g-PAA network may have mitigated the entanglement of the polymeric chains, beneficial to the enhancement of the water absorbency. The excessive LA would act as an additional cross-linking point in the polymeric network because the –OH groups on the LA surface could react with AA, which causes the elasticity of polymer chains to decrease and the water absorbency to decrease [38]. In spite of this, the water absorbency of WB-g-PAA/LA (10 wt%) was still greater than that of WB-g-PAA, which indicates that the incorporation of LA would effectively increase the water absorbency and reduce the production cost.

### 3.6. Evaluation of properties

#### 3.6.1. Studies of swelling kinetics in distilled water

**Figure 7.**
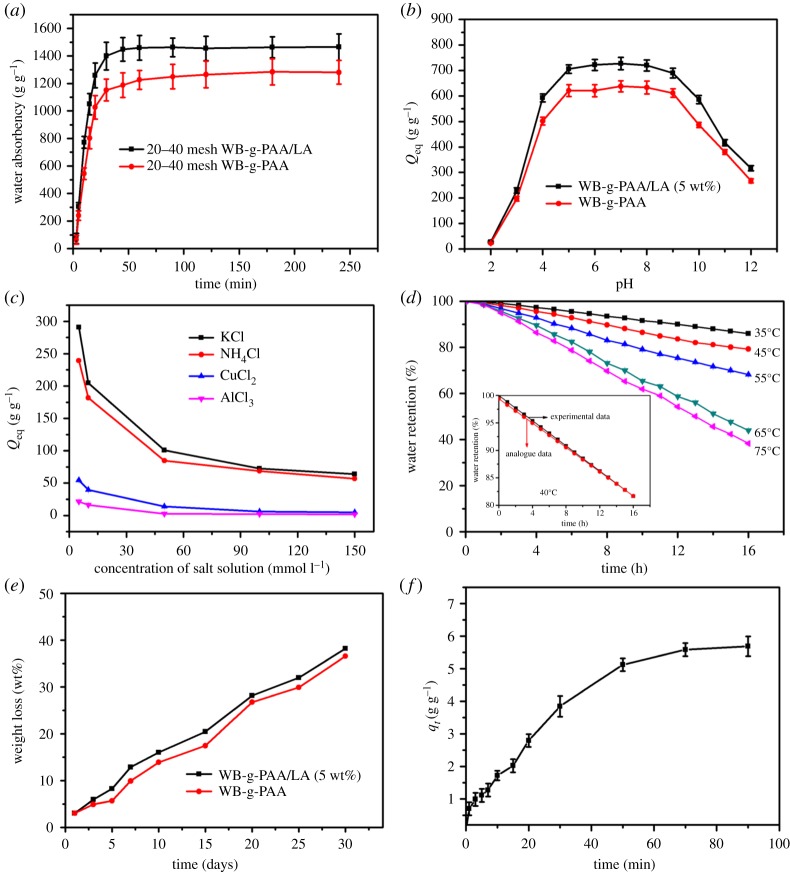
Evaluation of properties: (*a*) swelling kinetics in distilled water, (*b*) swelling behaviour at various pH values, (*c*) swelling behaviour in salt solutions, (*d*) water retention at different temperatures, (*e*) degradation in the soil supernatant and (*f*) urea loading process.

Figure 7*a* shows the swelling kinetics curves of WB-g-PAA/LA (5 wt%) and WB-g-PAA in distilled water (see detail section, electronic supplementary material, S1.1). The water absorbency sharply increased from 0 to 15 min, and the water absorbency of WB-g-PAA/LA (5 wt%) and WB-g-PAA can reach 1050 and 803 g g^−1^ after 15 min, respectively. For the same amounts, the swelling rate of WB-g-PAA/LA (5 wt%) was 1.3 times larger than that of WB-g-PAA within 15 min. The water absorbency rate of WB-g-PAA/LA (5 wt%) was up to 72 wt% of the equilibrium absorbency capacity, while that of WB-g-PAA was 63 wt%. From 15 to 60 min, the swelling kinetics curves slowly increased, which contributed to the equilibrium absorbency. Subsequently, the curves of the superabsorbent composites remained almost constant from 60 to 240 min. These results indicated that the incorporation of 5 wt% LA could accelerate the penetration of water and improve the water absorbency capability. This phenomenon was in conformity with the SEM images of WB-g-PAA and WB-g-PAA/LA (5 wt%).

#### 3.6.2. Studies of swelling behaviour at various pH levels

In practical applications, the degree of acidity or alkalinity has a significant impact on the water absorbency levels of superabsorbent composites [39]. In this work, the swelling behaviours of both WB-g-PAA/LA (5 wt%) and WB-g-PAA were studied in solutions of various pH levels, ranging from 2 to 12. Because ionic strength can obviously influence the water absorbency levels of superabsorbent composites, no additional ions (through buffer solution) were added to the medium when setting the pH. As can be seen in figure 7*b*, although WB-g-PAA/LA (5 wt%) had a greater water absorbency level than that of WB-g-PAA, they had similar swelling curves in various pH solutions. The equilibrium swelling capability of the samples continuously increased as the pH value increased from 2 to 4 and decreased as the pH value increased from 10 to 12. When the pH value was between 5 and 9, the equilibrium swelling capability remained roughly constant. Most of the carboxylate groups (−COO^−^) were protonated under acidic conditions (pH < 5), which weakened the repulsive forces among −COO^−^ groups. Consequently, the network structure of the superabsorbent composites tended to shrink, and the water absorbency decreased. When pH values were greater (5 < pH < 9), more carboxylate groups were ionized and the number of −COO^−^ groups increased, which caused the electrostatic repulsion to strengthen between the charged sites (−COO^−^), making the superabsorbent composites swell further [40]. In an extremely basic medium (pH > 9), the charge-screening effect occurred owing to the interaction between the −COO^−^ groups and excess Na cations in the swelling media, which hindered the repulsion efficiency and caused the water absorbency to decrease [41].

#### 3.6.3. Studies of swelling behaviour in salt solutions

It is necessary to clarify the effects of the external solution, including its cation types and the salt concentration, on the swelling behaviours of the superabsorbent composites for their utilization [42]. In this section, chloride salt solutions, including KCl, NH_4_Cl, CuCl_2_ and AlCl_3_, were employed as swelling media to investigate the effects of cation types and the salt solution concentration on the water absorbency of WB-g-PAA/LA (5 wt%). As shown in figure 7*c*, the swelling behaviour of the superabsorbent composites was affected by cation types and salt concentration. The water absorbency decreased as the concentrations of these salt solutions increased. Additionally, in the presence of different cations, the water absorbency levels of WB-g-PAA/LA (5 wt%) also followed the order K^+^ > NH_4_^+^ > Cu^2+^ > Al^3+^ at the same concentration. From a literature review [43], the differences could be mainly attributed to osmotic pressure and cross-linking density. The cations in the salt solutions were able to force the osmotic pressure between the external solution and the network of superabsorbent composites to reduce to different levels. The multivalent cations (Cu^2+^ and Al^3+^) form complexes with the carboxylate groups and affect the network structure in superabsorbent composites, leading to a high ionic strength. The ionic strength can decrease the water absorbency of the superabsorbent composite. In contrast with the CuCl_2_ solution, the ionic strength of the AlCl_3_ solution at the same concentration was greater, thus the water absorbency was also lower than that of CuCl_2_ solution. However, the smaller the monovalent cation's radius, the greater is the water absorbency. The radii of K^+^ and NH_4_^+^ are 1.33 and 1.42, respectively (in figure 7*c*), and the order of water absorbency capability in chloride salt solutions was K^+^ > NH_4_^+^. A similar result was reported for poly(aspartic acid) [44].

#### 3.6.4. Water-retention studies at different temperatures

The water-retention capabilities of superabsorbent composites play important roles in the growth of agriculture [45]. In this study, the water-retention capability of WB-g-PAA/LA (5 wt%) was determined by heating tests at 35, 45, 55, 65 and 75°C. As shown in figure 7*d*, the water-retention curves of WB-g-PAA/LA (5 wt%) displayed a continuous decreasing trend over time. When the temperature was lower than 55°C, WB-g-PAA/LA (5 wt%) exhibited a higher water-holding capability. Within 16 h, WB-g-PAA/LA (5 wt%) retained about 86%, 79%, 68%, 44% and 38% of water at 35°C, 45°C, 55°C, 65°C and 75°C, respectively. Compared with previous reports, [46,47], WB-g-PAA/LA (5 wt%) exhibited a similar water-retention capability, which indicates it can be further used as an effective water-retention agent for herbal medicine growth in arid soils. In addition, the water-retention rate equation followed a zero-order reaction based on the rate equation and the water-retention curve, [48] demonstrating that *WR* (%) and *t* had a linear relationship. The *WR* (%) could be calculated by the following equation:

3.1 $$[WR(\%)]=100-k_{n}t,$$

where *WR* (%) represents the percentage of water retention and *k_n_* represents the rate constant of water retention. The rate constant at different temperatures was *k*_35_* = *0.9074 min^−1^, *k*_45_* = *1.3980 min^−1^, *k*_55_* = *2.0744 min^−1^, *k_65 _= *3.6436 min^−1^ and *k_75 _= *3.9986 min^−1^. Using the Arrhenius equation (*k = Ae^−Ea/RT^*), the *k_n_* could be calculated by the following equation:

3.2 $$k_{n}=8.2428\times 10^{5}\text{exp}\left(\frac{-E_{a}}{RT}\right),$$

where the apparent activation energy (*E*_a_) was 35.11 kJ mol^−1^. Equation (3.2) revealed that the water-retention time of the WB-g-PAA/LA superabsorbent composite would be longer when the temperature was lower. The whole water-retention process is an endothermic process. By placing *T*_40_* *= 313.15 K into equation (3.2), the parameter *k*_40_* *= 1.1466 min^−1^. The *WR* (%) equation was obtained as follows:

3.3 $$[R_{n}]=100-1.1466t.$$

Based on the experimental data of the WB-g-PAA/LA superabsorbent composite at 40°C (313.15 K), the water-retention capacity is shown in figure 7*d*. The following experimental equation was obtained:

3.4 $$[R_{n}]=99.4156-1.1085t.$$

The comparison of equation (3.3) with equation (3.4), which were coincident with each other, indicated that equation (3.2) for the WB-g-PAA/LA superabsorbent composite may be applicable in the experimental range.

#### 3.6.5. Degradation studies in the soil supernatant

The superabsorbent composites immersed in the soil supernatant were monitored for one month. The result shows that the percentage weight loss of the superabsorbent composites is lower at the beginning but it became higher continuously over time. Moreover, the weight loss of WB-g-PAA/LA (5 wt%) is higher than that of WB-g-PAA, as in figure 7*e*. The percentage weight loss of WB-g-PAA/LA (5 wt%) and WB-g-PAA is 13 wt% and 10 wt% after 7 days, respectively. After 30 days, WB-g-PAA/LA (5 wt%) degraded to 38 wt%, but WB-g-PAA to 36 wt%. The reason may be that the superabsorbent composites are not completely swollen in the early stages, which is not conducive for microorganisms entering into the polymeric network. The microorganisms can attack more effectively and degrade the superabsorbent composites when the superabsorbent composites obtain the maximum swelling. The incorporation of 5 wt% LA can be helpful for improving the swelling of superabsorbent composites, which promotes the entry of microorganisms in the soil supernatant into polymeric network, leading to the rupture of polymeric chains [49].

#### 3.6.6. Studies of the urea loading process

The urea loading curve for WB-g-PAA/LA (5 wt%) is presented in figure 7*f*. It can be seen that the urea loading curve exhibits an upward trend within 60 min. Later, the urea loading curve flattens. The flattening time appeared to coincide with the time needed to achieve the maximum water absorbency in distilled water. The reason may be due to the following interpretation. Urea is a neutral molecule and an aqueous solution of urea may not affect the electrostatic repulsive forces of the −COO^−^ groups on superabsorbent composite chains. When WB-g-PAA/LA (5 wt%) was immersed in a urea solution, more urea molecules entered into the superabsorbent network with the water. After drying, these urea molecules were left in the network [50]. Thus, the urea loading depends on the degree of swelling of WB-g-PAA/LA in distilled water. In this section, the whole loading process (see detail section, electronic supplementary material, S1.5) for urea onto WB-g-PAA/LA (5 wt%) was analysed by a pseudo-first-order kinetic model, pseudo-second-order model and an intra-particle diffusion model, and the correlation coefficient values (*R*^2^) were 0.9803, 0.9189 and 0.9677, respectively (table 1), which revealed that the loading process for urea could fit the pseudo-first-order kinetic model because the correlation coefficient values of the pseudo-first-order kinetic model was closer to 1. Moreover, the calculated *Q*_e_ values for pseudo-first order were in agreement with the experimental *Q*_e_ values. These results further indicated that the urea loading process onto WB-g-PAA/LA (5 wt%) followed a pseudo-first-order kinetic model.

**Table 1.** Urea loading process parameters of WB-g-PAA/LA (5 wt%) in 5 mg ml^−1^ urea aqueous solutions.

pseudo-first-order model			pseudo-second-order model			intra-particle diffusion model	
*K*_1_ (g g^−1^ min^−1^)	*Q*_e_ (g g^−1^)	*R*^2^	*K*_2_ (g g^−1^ min^−1^)	*Q*_e_ (g g^−1^)	*R*^2^	*K_i_* (g g^−1^ min^−1^)	*R*^2^
0.03	5.84	0.9803	0.48×10^−2^	7.50	0.9189	0.68	0.9677

### 3.7. Application studies

#### 3.7.1. *Glycyrrhiza uralensis* Fisch growth

The effects of superabsorbents on the growth and yield of crops have been studied [51,52]. However, limited studies have focused on the growth of traditional medicinal herbs with great medicinal value. *Glycyrrhiza uralensis* is a traditional medicinal herb with great medicinal value that is mainly distributed in arid and semi-arid areas, which could also play an important role in soil and ecological conservation in desert areas. Wild resources have become less and less due to extensive digging. Here, with the goal of studying the effects of WB-g-PAA/LA (5 wt%) on the germination and growth rates of *G. uralensis*, different amounts of WB-g-PAA/LA (5 wt%) were investigated (see detail section, electronic supplementary material, S1.7). As the amount of WB-g-PAA/LA (5 wt%) increased in the soil, the germination and growth rates of *G. uralensis* increased (table 2). After 25 days, the germination and growth rates of *G. uralensis* in untreated soil (control) and soil with 1.5 wt% of WB-g-PAA/LA (5 wt%) were compared, and the latter had a striking effect on the plant height, plant weight and root length of *G. uralensis* (table 2). The WB-g-PAA/LA (5 wt%) superabsorbent composite had good water absorbency and retention capabilities, which could supply water to *G. uralensis*, promoting germination and growth. Based on the initial study results, WB-g-PAA/LA (5 wt%) has potential applications in the growth of traditional medicinal herbs, especially in the growth of *G. uralensis*.

**Table 2.** The germination rate and growth of *G. uralensis* in soil containing different amounts of WB-g-PAA/LA (5 wt%). Values represent the mean of three replicates.

WB-g-PAA/LA content (wt%)	germination rate (%)^a^	plant height (cm)^b^	root length (cm)^b^	plant weight (g)^b^
WB-g-PAA/LA (0 wt%)	63.3	4.9	17.3	0.29
WB-g-PAA/LA (0.5 wt%)	66.7	5.2	19.1	0.34
WB-g-PAA/LA (1.0 wt%)	73.3	5.6	20.4	0.36
WB-g-PAA/LA (1.5 wt%)	76.7	6.9	21.7	0.41

^a^Data at 14 days after sowing *G. uralensis* Fisch.

^b^Data at 25 days after sowing *G. uralensis* Fisch.

#### 3.7.2. Effects on urea release in soil

**Figure 8.**
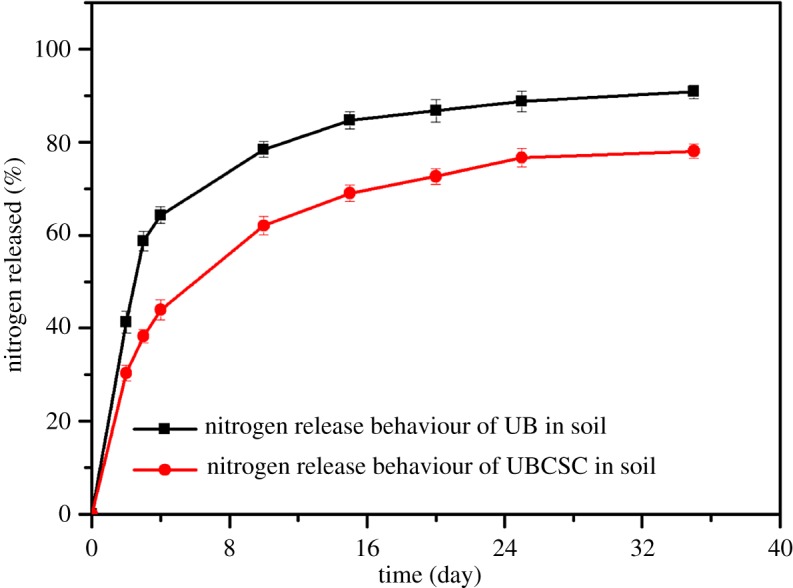
Effects on urea release behaviour in soil.

At room temperature, the release rates of UBCSC and UB (control) were monitored in the soil. As presented in figure 8, 64.3 wt% of nitrogen was released within 4 days for the UB, while 98.5 wt% of nitrogen was released within 12 hours from untreated urea in the reported literature [53]. Apparently, the UB improved the urea utilization efficiency in contrast with untreated urea. However, the UBCSC released 62 wt% even after 10 days, which was less than the UB. The WB-g-PAA/LA (5 wt%) superabsorbent composite played an important role in retarding urea nitrogen release. When the UBCSC was buried in the soil, the outer WB-g-PAA/LA (5 wt%) superabsorbent composite may have absorbed the water in soil, resulting in the transformation into hydrogel. Then, the free water in WB-g-PAA/LA (5 wt%) may have been transferred to the UB, which would cause the soluble urea to be dissolved. Consequently, the dissolved urea spread out from the UB and was absorbed by WB-g-PAA/LA (5 wt%). Finally, the urea was released into soil through the dynamic exchange of free water. In this way, the WB-g-PAA/LA (5 wt%) superabsorbent composite regulated the release of urea and mitigated its burst release effect [54].

## 4. Conclusion

A low-cost and eco-friendly WB-g-PAA/LA superabsorbent composite was successfully prepared through free-radical graft co-polymerization in an aqueous solution using KPS as the initiator, MBA as the cross-linker and LA as the inorganic filler. FTIR spectra revealed that the WB and LA successfully participated in the co-polymerization. Incorporating 5 wt% of LA in WB-g-PAA would result in the final superabsorbent composite becoming loose and rough, which greatly improved the swelling property (1050 g g^−1^) within 15 min compared with the neat superabsorbency (803 g g^−1^). Moreover, the water-retention rate process followed a zero-order reaction, the reaction rate constant is 8.2428 × 10^5^exp(−*E*_a_*/RT*) and the apparent activation energy (*E*_a_) is 35.11 kJ mol^−1^. Importantly, the urea loading onto WB-g-PAA/LA (5 wt%) was outstanding, and the urea loading process of WB-g-PAA/LA (5 wt%) followed the pseudo-first-order kinetic model. A degradation rate of 38 wt% was achieved when the composite was immersed in soil supernatant for 30 days. In addition, approximately 64.3 wt% of nitrogen was released within 4 days from the UB, while approximately 62 wt% was released after 10 days for the UBCSC. Furthermore, soil with WB-g-PAA/LA (5 wt%) had considerable effects on the germination rate and early growth of *G. uralensis*. Thus, the developed WB-g-PAA/LA superabsorbent composite could be used as a suitable additive in modern *G. uralensis* growth. The novel method also exhibited a promising use of natural resources, such as WB and LA, in new value-added applications.

## Supplementary Material

Preparation of a Low-cost and Eco-friendly Superabsorbent Composite Based on Wheat Bran and Laterite for Potential Application in Chinese Herbal Medicine Growth

## Data accessibility

The datasets supporting this article have been uploaded as part of the electronic supplementary material.
