# Supplementary material for: Preparation of a low-cost and eco-friendly superabsorbent composite based on wheat bran and laterite for potential application in Chinese herbal medicine growth
Source: R Soc Open Sci. 2018 May 23;5(5):180007. doi: 10.1098/rsos.180007 (PMC5990732; doi:10.1098/rsos.180007)
Supplement: Preparation of a Low-cost and Eco-friendly Superabsorbent Composite Based on Wheat Bran and Laterite for Potential Application in Chinese Herbal Medicine Growth [file rsos180007supp1.doc]

Preparation of a Low-cost and Eco-friendly Superabsorbent Composite Based on Wheat Bran and Laterite for Potential Application in Chinese Herbal Medicine Growth

**Jiande Gao^a,b^, Jin Liu ^a^, Hui Peng^a^, Yaya Wang ^a^, Sha Cheng ^a^, Ziqiang Lei^a*^**

*^a^**Key Laboratory of Eco-Environment-Related Polymer Materials of Ministry of Education, Key Laboratory of Polymer Materials of Gansu Province, College of Chemistry and Chemical Engineering, Northwest Normal University, Lanzhou 730070, China. E-mail:* *leizq@nwnu.edu.cn*

*^b^College of Pharmacy, Gansu University of Traditional Chinese Medicine, Lanzhou 730000, China.*

Experimental details

**1 Evaluation of properties**

**1. 1 Measurements of the equilibrium water absorbency and swelling kinetics**

The water absorbency of the superabsorbent composites was determined using a conventional gravimetric method: A weighed dry sample (*W_0_* = 0.05 ± 0.001 g) with average particle sizes between 20 and 40 mesh were immersed in 300 mL distilled water or 0.9 wt% of NaCl solution at room temperature for 8 h to reach the swelling equilibrium. The fully swollen samples were filtered through a 100-mesh screen to remove the surface water and weighed (*W_1_*). The equilibrium water absorbency (*Q_eq_*, g/g) was determined according to Eq. (1):

*Q_eq_* = *(W_1_–W_0_)/W_0_*, (1)

where *W_0_* and *W_1_* indicate the weights of the dry and water-swollen sample, respectively.

The swelling kinetics of the superabsorbents were measured according to the method used by Ashraf and coworkers.^1^ Approximately 0.05 g of sample was mixed with 300 mL of distilled water. The swollen samples were filtered and weighed at different intervals (*t*; 3, 5, 3, 10, 15, 20, 30, 45, 60, 90, 120, 180, and 240 min), and the water absorbency levels of the samples were calculated using Eq. (1).

**1. 2 Measurements of water absorbency at various pH levels**

The method of determining water absorbency levels at various pH levels was similar to that of a previous report.^2^ A series of solutions with different pH levels were prepared and adjusted using NaOH (pH = 13.0) and HCl (pH = 1.0) solutions to achieve the desired pH. The pH values of the solutions were precisely measured using a pH meter. The equilibrium water absorbency values of the samples (0.05 ± 0.001 g) in various pH solutions were determined using Eq. (1)*.*

**1. 3 Measurements of water absorbency in salt solution**

KCl, NH_4_Cl, CuCl_2_ and AlCl_3_ were used as water absorbency media at different concentrations (5, 10, 50, 100 and 150 mmol/L). Then, approximately 0.05 g of product was added to 300 mL of each chloride salt solution. The water absorbency capabilities in the salt solutions were determined in the same way, as described in Eq. (1).

**1. 4 Water-retention behavior**

The determination of the water retention was carried out according to the following procedure: About 0.05 g of dry sample was immersed in distilled water at room temperature to reach the swelling equilibrium. The swollen gels were removed and hung for 10 min in a tea bag to eliminate excess water. They were then weighed (*W_0_*), baked in an oven at 35°C, 45°C, 55°C, 65°C and 75°C, independently, and weighed accurately at certain time intervals (*W_t_*). The percentage water retention (*WR%*) of the superabsorbent composites was calculated using Eq (2):

, (2)

where *W_d_* indicates the weight of the dry sample.

**1. 5 Evaluation of the urea loading process**

The urea loading process was performed at room temperature and was similar to that of a previous report.^3^ Approximately 0.05 g of sample was immersed in 300 mL of urea solution of 5 mg/mL without stirring, at desired *t* (1, 3, 5, 7, 10, 15, 20, 30, 50, 70 and 90 min), the samples in urea solution were removed and dried at 50°C for 6 d to constant weights. The experiment was performed at room temperature. The loading amount of urea, *q_t_* (g/g), at variable times was calculated using Eq. (3):

*q_t_* = *(m_t_ − m_1_)/m_1_*, (3)

where *m_t_* and *m_1_* indicate the weights of loaded urea dry gels at any time (*t*) and unloaded urea dry gels (g), respectively.

**1. 6 Data analysis of the urea loading process**

To further analyze the mechanism of the urea loading process for the WB-g-PAA/LA (5 wt%) superabsorbent composite, the following three models were used: the pseudo-first-order model [Eq. (4)], the pseudo-second-order model [Eq. (5)], and the intraparticle diffusion model [Eq. (6)]. These models are expressed by the following equations:

(4)

(5)

, (6)

where$Q_{e}$ (g/g) and $Q_{t}$ (g/g) represent the loading capacity at equilibrium and at any *t* (min), respectively; $k_{1}$ (g g^−1^ min^−1^), $k_{2}$ (g g^−1^ min^−1^) and $k_{i}$ (g g^−1^ min^−0.5^) represent the rate constants of the pseudo-first-order, the pseudo-second-order and the intraparticle diffusion kinetic models, respectively; and *C* indicates the constant of the intraparticle diffusion model. The plot of $\text{ln}(Q_{e}-Q_{t})$versus $t$produces a straight line, from which $k_{1}$ and $Q_{e}$can be determined using the slope and intercept. In Eq. (5), the values of $Q_{e}$ and $k_{2}$ could be calculated from the slope and intercept of the plot of $t/Q_{t}$against $t$. In Eq. (6), the plot of $Q_{t}$ vs $t^{0.5}$ resulted in a linear relationship with a slope of $k_{i}$ and an intercept of *C* when the urea loading process follows the intraparticle diffusion process.

**1.7** ***Glycyrrhiza uralensis* Fisch (*G. uralensis*) growth**

To study the effect of the WB-g-PAA/LA (5 wt%) on *G. uralensis* growth, the following procedure was performed: Firstly, the dry soil (below 20 mesh) was placed into plastic cups having four holes each of a 0.1-mm diameter. The depth of the soil was 12 cm in each cup. The soil had been collected from Lanzhou, which is located in northwestern China and enjoys a semi-arid climate. The properties of the soil have been reported in previous studies.^4-5^ Then, healthy *G. uralensis* seeds were selected and immersed in concentrated sulfuric acid for 30 min to break dormancy because of the hardness of *G. uralensis* seeds. Subsequently, *G. uralensis* seeds were rinsed thoroughly with distilled water, and placed into the plastic cups (10 seeds/cup), and covered with the mixture of dry soil (below 20 mesh) and WB-g-PAA/LA (5 wt%) superabsorbent composite. Each plastic cup was slowly irrigated with water. Thereafter, no water was applied. The numbers of germinated seeds were noted for up to two weeks to determine the germination rates. The experiment was complete when seedlings wilted for the first time. The averages of plant heights and shoot lengths were determined using a ruler. The plant weight was determined after transferring the samples into an oven at 70°C for 24 h. Germination rates of the seeds were calculated using Eq. (7):

Germination rate (%) = number of germinated seeds/total seeds × 100%. (7)
In all cases, three parallel samples were measured, and the final results were averaged.

**2. Characterization**

Scanning electron microscopy (SEM) and Elemental map (EM) were examined using a ULTRA Plus SEM instrument (Carl Zeiss AG) system at an acceleration voltage of 5 kV. And the superabsorbent composite sample was coated with gold film before measurement. Fourier transform infrared (FTIR) spectra were recorded using a FTIR-FTS3000 (resolution: 1.0 cm^−1^, signal to noise ratio: 15,000: 1(P-P) in 400–4000 cm^−1^ wavenumber range. The samples were mixed perfectly with KBr and compressed into KBr thin pellets. The thermogravimetric analyses (TGA)/differential thermal analysis (DTG) was obtained by an America TA Company Instruments (TGA-Q100) under nitrogen at a heating rate of 10 °C/min until reached 800 °C. The Rigaku D/Max-2400 diffractometer was used to analyze the X-ray patterns (XRD) of samples at 2 theta between 10° and 80°, at a scanning speed of 5°/min.

**3. Mechanism of superabsorbent composite formation**

The WB-g-PAA/LA was prepared by graft copolymerization of acrylic acid onto WB in the presence of initiator, crosslinking agents and powdery LA. The proposed mechanism for the grafting and chemically crosslinking reactions are shown in Scheme 1. Initially, the persulfate initiator is decomposed under heating to generate sulfate anion-radicals that abstract hydrogen atoms from the hydroxyl group of the polysaccharide (cellulose, starch containing in WB) to form alkoxy radicals on the substrate. Thereafter, these alkoxy radicals reacted with the monomer molecules, AA, which caused the graft chain to grow. Meanwhile, the polymer chains reacted with the cross-linker MBA and LA particles, during chain propagation. The copolymer is comprised of a crosslinked structure, which formed the network. Ma et al. has recently reported a similar proposed mechanism.^6^


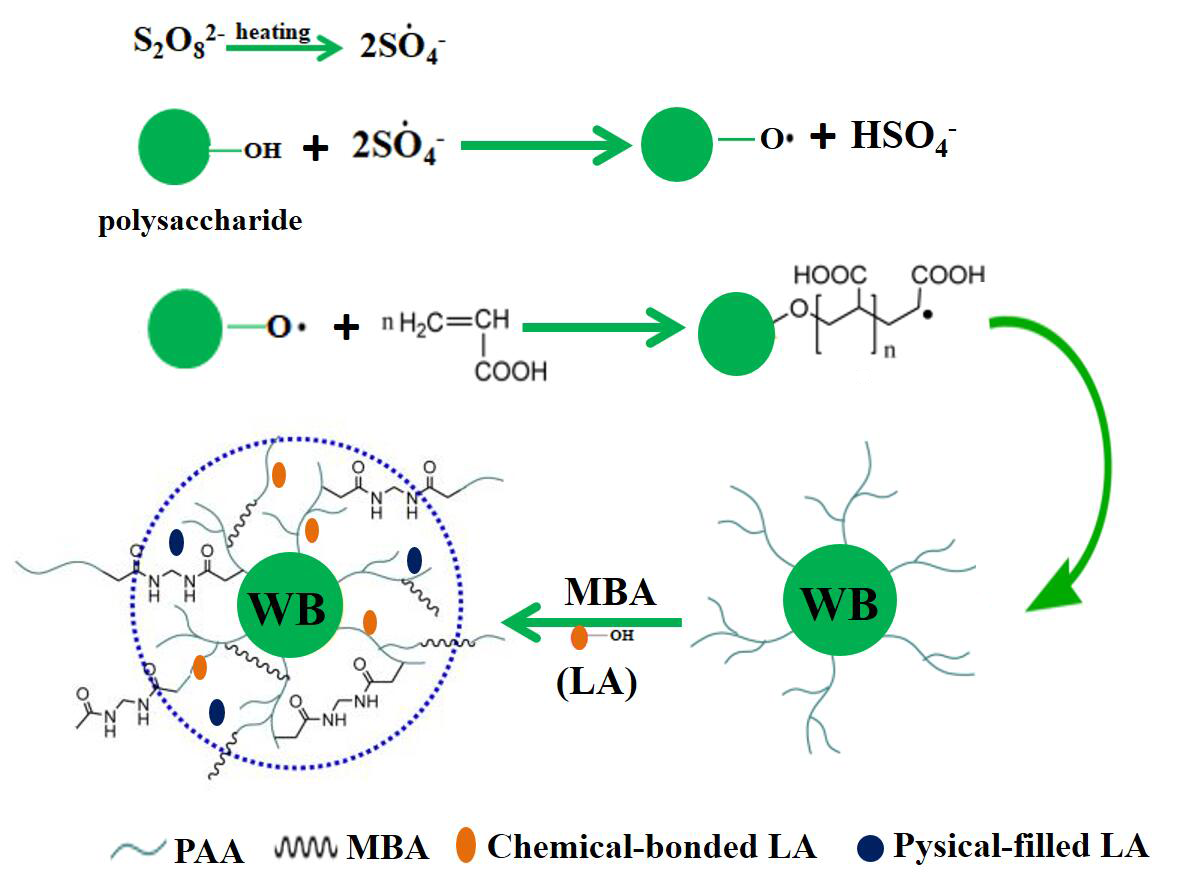


**Scheme 1**. Proposed reaction mechanism for preparation of WB-g-PAA/LA.

**References**

1. M.U. Ashraf, M.A. Hussain, G. Muhammad, M.T. Haseeb, S. Bashir, S.Z. Hussain, I. Hussain, *Int. J. Biol. Macromol.*, 2017, 95, 138–144.
2. E.K. Feng, G.F. Ma, Y.J. Wu, H.P. Wang, Z.Q. Lei, *Carbohydr. Polym.*, 2014, 111, 463–468.
3. Y. Zhang, F. Wu, L. Liu, J.M. Yao, *Carbohydr. Polym.*, 2013, 91, 277–283.
4. B.L. Ni, M.Z. Liu, S.Y. Lü, L.H. Xie, Y.F. Wang, *J. Agric. Food Chem.*, 2011, 59, 10169–10175.
5. B.L. Ni, S.Y. Lü, M.Z. Liu, *Ind. Eng. Chem. Res.*, 2012, 51, 12993–13000.
6. G.F., Ma, Q.,Yang, F. T., Ran, Z.B., Dong, Z.Q., Lei, *Appl. Clay Sci.,* 2015, 118, 21-28.
